# ﻿Two new species of Nectriaceae (Hypocreales, Sordariomycetes) from Yunnan, China

**DOI:** 10.3897/mycokeys.108.130098

**Published:** 2024-09-10

**Authors:** Hua Zheng, Xinwen Dai, Haiyan Li, Zefen Yu

**Affiliations:** 1 Medical School, Kunming University of Science and Technology, Kunming, Yunnan, 650500, China Kunming University of Science and Technology Kunming China; 2 Laboratory for Conservation and Utilization of Bio-resources, Key Laboratory for Microbial Resources of the Ministry of Education, Yunnan University, Kunming, Yunnan, 650032, China Yunnan University Kunming China

**Keywords:** Air-borne or soil-borne fungi, fungal diversity, Hypocreales, phylogeny, taxonomy

## Abstract

Nectriaceae is a highly diverse family, and members have a worldwide distribution, particularly in warm temperate to tropical regions. During the survey of fungal diversity in different habitats in Yunnan province, China, two new species isolated from soil and air respectively, namely *Atractiumyunnanense***sp. nov.** and *Nalanthamalaxishuangbannaensis***sp. nov.**, were proposed based on morphological comparisons and the multi-gene phylogenetic analyses of combined ITS, LSU, *rpb*2, and *tub*2 sequence data. Phylogenetically, both species clustered in a monophyletic clade within Nectriaceae with strong support. *A.yunnanense* is characterized by synnematous conidiophores, pale olivaceous-green, clavate to oblong-ellipsoidal, multi-septate conidia, and pale olivaceous-green chlamydospores. *N.xishuangbannaensis* has acremonium-like or penicillium-like conidiophores and either obovate or ellipsoidal, cylindrical or fusiform conidia. Full descriptions, illustrations, and a phylogenetic tree showing the phylogenetic position of the two new species were provided.

## ﻿Introduction

Nectriaceae was originally established based on *Nectria* (Fr.) Fr. and assigned in Hypocreales (Tulasne and Tulasne 1865). Thereafter, the taxonomy of Nectriaceae has undergone several revisions (Dennis 1960; Rossman et al. 1999; Lombard et al. 2015). This family is characterized by uniloculate and verrucose ascomata by reaction in KOH and lactic acid, and unitunicate, 2–8-spored asci with globose, ellipsoid to long-fusiform ascospores. Additionally, it is associated with phialidic asexual morphs producing amerosporous to phragmosporous conidia (Lombard et al. 2015). In a recent study, Perera et al. (2023) proceeded to update DNA sequence-based phylogeny for the order Hypocreales, and accepted 77 genera into Nectriaceae, including *Atractium* Link and *Nalanthamala* Subram. Nectriaceae is a highly diverse group, and members have a cosmopolitan distribution, particularly in the warm temperate and tropical regions (Rossman et al. 1999; Chaverri et al. 2011; Lombard et al. 2015). Particularly, this family has ecological and economic significance. The majority of the species are soil-borne saprobes or weak to virulent plant pathogens, and include important plant pathogens of economically important plants worldwide, such as *Ilyonectria* species and *Fusarium* species (Jayawardena et al. 2019a, 2019b). Moreover, several species have also been reported as important opportunistic pathogens of humans (Chang et al. 2006; Guarro 2013; Köhler et al. 2015, 2017; Hoenigl et al. 2021). For instance, a patient with acute myelogenous leukemia experienced a disseminated cerebellar *Fusarium* infection, which magnetic resonance imaging and a CT scan revealed to be a left cerebellar focus of *Fusariumsolani* (Vincent et al. 2003).

*Atractium* was introduced by Link (1809) with *A.stilbaster* Link as the type species. So far, there are 28 epithets for *Atractium* as listed in the Index Fungorum (https://www.indexfungorum.org; retrieval in June 2024). Of them, five species were accepted in the genus, while the placement of the other 23 species remains uncertain due to disagreement with the emended generic diagnosis or an inability to locate authentic material (Gräfenhan et al. 2011; Bao et al. 2023). Gräfenhan et al. (2011) conducted a comprehensive phylogenetic reassessment of nectriaceous fungi, and accepted three asexual species *A.crassum* (Wollenw.) Seifert & Gräfenhan, *A.holubovae* (Seifert, S.J. Stanley & K.D. Hyde) Seifert and *A.stilbaster* in *Atractium*. In a recent study, a sexual morph species *Varicosporellaaquatica* Lechat & J. Fourn. was transferred to *Atractium*, namely *A.aquatica* (Lechat & J. Fourn) D.F. Bao, K.D. Hyde & Z.L. Luo, and a new species *A.fusiformis* D.F. Bao, K.D. Hyde & Z.L. Luo was introduced based on morphological and phylogenetic analysis (Bao et al. 2023). The asexual morph of *Atractium* is characterized by synnematous conidiophores, monophialidic, subulate conidiogenous cells with septate, clavate, obovoid or gently curved conidia, and its sexual morphs fit well with the generic concepts of Nectriaceae (Bao et al. 2023). Currently, single *Atractium* species (*A.fusiformis*) was reported as saprobe from China among the five accepted species.

*Nalanthamala* was proposed for *N.madreeya* Subram. and characterized by mononematous or aggregated conidiophores, singly or in whorls produced phialides, and elliptical to oval or lenticular conidia arranged in chains (Subramanian 1956). Gams (1975) placed *Fusidiumsquamicola* Berk. & Broome in *Nalanthamala* based on morphological resemblance of conidia, namely *N.squamicola* (Berk. & Broome) W. Gams (Gams 1975). Schroers et al. (2005) demonstrated that *Nalanthamala* belongs in Nectriaceae using LSU rDNA sequences, and accepted three species, including *N.diospyri* (Crandall) Schroers & M.J. Wingf., *N.psidii* (Sawada & Kurosawa) Schroers & M.J. Wingf., and *N.vermoesenii* (Biourge) Schroers. Subsequently, Rossman et al. (2013) proposed *Nalanthamala* over *Rubrinectria* Rossman & Samuels, and included *N.olivacea* (Seaver) Rossman in this genus. However, Crous et al. (2021) transferred *N.squamicola* to the newly established genus *Caespitomonium* Crous in Bionectriaceae, named after *C.squamicola* (Berk. & Broome) Crous. Currently, there are five species in this genus *Nalanthamala* (Wijayawardene et al. 2020; Crous et al. 2021). Interestingly, the known *Nalanthamala* species are also associated with wilt and blight diseases of several economically important crops (Schroers et al. 2005; Rossman et al. 2013). However, a single species, *N.psidii*, has been recorded from China (Schroers et al. 2005).

Southwest China is one of the high biodiversity hotspots in the word (Myers et al. 2000; Orme et al. 2005; Mi et al. 2021). Particularly, there is a high fungal diversity in Yunnan province (Feng and Yang 2018; Zheng et al. 2021b). In the last decade or so, our team discovered many new fungal species during the investigation of fungal diversity in different habitats in Yunnan (Zheng et al. 2019, 2020, 2021a, 2022; Qiao et al. 2020, 2021; Yu et al. 2022), which increases knowledge of this important ecological area. In this study, two new taxa in Nectriaceae were discovered from Yunnan, and described as new species of *Atractium* and *Nalanthamala*, namely *A.yunnanense* sp. nov. and *N.xishuangbannaensis* sp. nov., based on morphological characteristics and multi-gene phylogenetic analysis.

## ﻿Materials and methods

### ﻿Sample collection, fungal isolation and morphological characterization

Soil samples were collected from Huize county, Yunnan province. Samples were preserved in sterile plastic bags, labeled and transported to the laboratory at 4 °C. The dilution coating method was used to isolate fungal strains from soils as described by Lv et al. (2022). The fungi in air located at Xishuangbanna, Yunnan province, were collected by means of the MAS-100 ISO MH Microbial Air Sampler (Merck Millipore, Germany). Ten liters of air flowed through the surface of each Rose Bengal agar (Guangdong Huankai Microbial Sci and Tech, China) plate which placed in the air sampling equipment. After incubation at 25 °C for 5 days, representative colonies were picked up with a sterilized needle and maintained on potato dextrose agar (PDA; 200 g potato, 20 g dextrose, 18 g agar, 1000 ml distilled water) plates. The pure strains were incubated on PDA and cornmeal agar (CMA; 20 g cornmeal, 18 g agar, 1000 ml distilled water) for observing morphological characteristics of colonies. Microscopic characteristics growing on CMA were examined and captured by an Olympus BX51 microscope connected to a DP controller digital camera, and sterile water was used as a mounting medium for microscopy.

The pure cultures were deposited in the Herbarium of the Laboratory for Conservation and Utilization of Bio-Resources, Yunnan University, Kunming, Yunnan, P.R. China (**YMF**), China General Microbiological Culture Collection Center (**CGMCC**), the Guangdong Microbial Culture Collection Center (**GDMCC**) and Japan Collection of Microorganisms (**JCM**). MycoBank numbers were obtained in MycoBank database (https://www.mycobank.org/) for the newly-described taxa.

### ﻿DNA extraction, PCR amplification and sequencing

Total DNA was extracted from fresh mycelia grown on PDA for 7 days, as described by Turner et al. (1997). The primer pairs ITS5/ITS4 (White et al. 1990), LR0R/LR7 (Vilgalys and Hester 1990), rpb2-5F2/rpb2-7CR (Liu et al. 1999), and TUB-2Fd/TUB4RD (Woudenberg et al. 2009) were used for amplification of the internal transcribed spacers (ITS), the large subunit nuclear ribosomal RNA gene (LSU), the RNA polymerase II second largest subunit gene (rpb2), and the beta-tubulin gene (*tub*2), respectively. The PCR amplifications were performed in 25 µl reaction volumes containing 1.0 µl DNA template, 1.0 µl of each forward and reverse primers, 12.5 µl 2 × Master Mix, and 9.5 µl ddH_2_O. The PCR products were confirmed on 1% agarose electrophoresis gels, and the positive products were sent to Tsingke Biotechnology Company (Kunming, China) for purification and sequencing. Newly obtained sequences were deposited in the GenBank database at the National Center for Biotechnology Information (**NCBI**) and the accession numbers are listed in Table 1.

**Table 1.** Species, strains and their corresponding GenBank accession numbers of sequences used for phylogenetic analyses.

Species	Strains	GenBank accession no.			
		ITS	LSU	*rpb*2	*tub*2
*Atractiumaquatica*	CBS126103^T^	KP192669	KP192671	–	–
	CBS138883	KP192668	KP192670	–	–
*Atractiumcrassum*	CBS 180.31^T^	KM231790	U88110	–	KM232049
*Atractiumfusiformis*	KUNCC22-12521	OP876729	OP875082	–	OQ025196
	KUNCC22-12523^T^	OP876725	OP875078	–	OQ025192
	KUNCC22-12452	OP876727	OP875080	–	OQ025195
*Atractiumstilbaster*	CBS 410.67^T^	KM231791	KM231654	–	KM232050
	CBS 783.85	KM231792	KM231655	–	KM232051
***Atractiumyunnanense***	**YMF 1.06524^T^**	**OM985710**	**PP915817**	**PP928787**	**PP928793**
	**H77**	**PP915812**	**PP915818**	**PP928788**	**PP928794**
	**H102**	**PP915813**	**PP915819**	**PP928789**	**PP928795**
*Cosmosporaaquatica*	MFLUCC13-0884^T^	NR_168211	MK828238	MN194021	–
*Cosmosporabutyri*	DAOM216335	JN942831	JN938895		–
*Cosmosporacymosa*	CBS 762.69^T^	NR_111605	NG_058891	HQ897778	–
*Fusariumcoffeatum*	CBS 635.76	MH861016	AY213706	KU604328	–
*Fusariumequiseti*	NL19-25004	MZ890491	MZ890346	MZ921701	–
*Fusariumincarnatum*	CBS 161.25	MH854830	MH866331	MN170381	–
*Fusariumproliferatum*	CBS 263.54	KM231815	KM231684	KM232383	–
	CBS 153.27	MH854910	MH866404	–	–
*Ilyonectriacapensis*	CBS 132815^T^	NR_152887	MH878251	KM232336	–
*Ilyonectriacoprosmae*	CBS119606	JF735260	KM515910	KM232338	–
*Nalanthamaladiospyri*	CBS 430.89	AY554209	AY554248	–	AY554228
*Nalanthamalaeleanorwilliamsiae*	BRIP 66236a	OQ917077	–	–	–
*Nalanthamalaolivacea*	CBS 102268	AY554219	AY554244	–	AY554238
*Nalanthamalapsidii*	CBS 687.97	AY554208	–	–	AY554227
	CBS 110184	AY554207	–	–	AY554226
	CBS 110188	AY554206	–	–	AY554225
*Nalanthamalavermoesenii*	CBS 110893^T^	AY554214	AY554246	–	AY554233
	CBS 137.24	AY554217	AY554260	–	AY554236
***Nalanthamalaxishuangbannaensis***	**YMF 1.05062^T^**	**PP915809**	**PP915814**	**PP928784**	**PP928790**
	**B413**	**PP915810**	**PP915815**	**PP928785**	**PP928791**
	**B425**	**PP915811**	**PP915816**	**PP928786**	**PP928792**
*Nectriabalansae*	CBS 124070	JF832652	JF832710	–	JF832907
	AR4635	JN995622	JN939838	–	JF832908
*Neonalanthamalagraminearum*	CGMCC3.25240^T^	OQ733285	–	OQ716735	OQ716739
	S2	OQ733286	–	OQ716736	OQ716740
	S4	OQ733287	–	OQ716737	OQ716741
*Neonectriaaquatica*	KUNCC22-12462^T^	OP876733	OP875087	–	OQ025197
*Neonectrialugdunensis*	CBS 250.58	KM515893	KM515938	–	–
*Neonectriaramulariae*	CBS 151.29	JF735313	AY677333	DQ789792	–
	CBS 182.36	JF735314	HM042435	DQ789793	JF735439
*Sarocladiumsummerbellii*	CBS 430.70	MH859780	MH871543	–	–

Notes: ^T^ = Ex-type; New isolates are in bold; The line “–” represents the absence of GenBank record.

### ﻿Sequence alignment and phylogenetic analysis

Initially, the newly-generated sequences were subjected to the GenBank nucleotide database (https://blast.ncbi.nlm.nih.gov/) using BLAST searches to determine closely related taxa, including *Atractium* species and *Nalanthamala* species. To further determine the phylogenetic position of these strains, a multi-locus phylogenetic analysis was performed based on ITS, LSU, *rpb*2 and *tub*2. Alignments of different loci, including the sequences obtained from this study and sequences downloaded from GenBank, were initially aligned with ClustalX v1.83 (Thompson et al. 1997). The resulting alignments were subsequently checked and refined using BioEdit v7.0.4.1 (Hall 1999). The concatenation of the four loci was processed with BioEdit, and converted to a NEXUS file using MEGA v10 (Kumar et al. 2018). The concatenated sequence matrix contained 3,064 nucleotide positions from the four loci (633 from ITS, 886 from LSU, 932 from *rpb*2, and 613 from *tub*2), and was uploaded to TreeBASE (https://www.treebase.org; submission number: S31491).

Maximum Likelihood (ML) and the Bayesian Inference (BI) methods were used for the phylogenetic construction in this study. The ML analysis was performed by RAxML v8.0.9 (Stamatakis 2006) using the GTR-GAMMA model with rapid bootstrap analysis, followed by 1,000 ultrafast bootstrap replicates to estimate bootstrap support values (BS). The BI analysis was conducted with MrBayes v3.2.2 (Ronquist et al. 2012) with NEXUS file. The jModelTest v2.0 (Posada 2008) was used to carry out statistical selection of best-fit models of nucleotide substitution using the corrected Akaike information criterion (AIC), and the estimated best-fit model was GTR+F+I+G4. Markov Chain Monte Carlo (MCMC) simulations were used for 1,000,000 generations with a sampling frequency of every 500^th^ generations. The initial 25% of sample trees were treated as burn-in and discarded. The remaining trees were used to calculate the Bayesian posterior probabilities (BP). The phylogenetic trees were visualized using FigTree v1.4.3 and subsequently edited in Adobe Photoshop.

## ﻿Results

### ﻿Phylogenetic analysis

**Figure 1.**
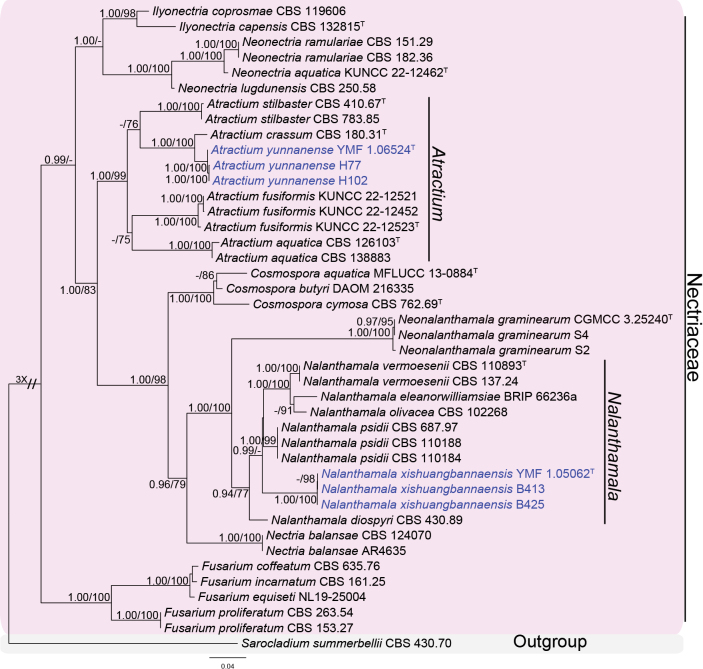
Phylogenetic tree inferred from a Maximum likelihood analysis based on a concatenated alignment of ITS, LSU, *rpb*2 and *tub*2 sequences of some representing species in Nectriaceae. The Bayesian posterior probabilities (BP) ≥ 0.9 and RAxML bootstrap support values (BS) ≥ 75% were shown at the nodes (BP/BS). Strains obtained in this study are shown in blue font. Ex-type strains are marked by a ^T^ after the strain number. The tree was rooted to *Sarocladiumsummerbellii* (CBS 430.70).

The concatenated dataset comprised 41 taxa (including our strains) representing eight genera in Nectriaceae (Hypocreales), with *Sarocladiumsummerbellii* (CBS 430.70) as the outgroup. The phylogenetic trees using ML and BI analyses were consistent and strongly supported in most branches. The topology of the phylogenetic tree is shown in Fig. 1, with maximum likelihood bootstrap support values (BS) ≥ 75% and Bayesian posterior probabilities (BP) ≥ 0.90 were shown at the nodes. In this tree, three isolates (YMF 1.06524, H77 and H102) clustered in a well-separated clade with a high support value (BP/BS = 1.00/100) and sister to *Atractiumcrassum* (CBS 180.31) with strong support (BP/BS = 1.00/100). The other three isolates (YMF 1.05062, B413 and B425) formed a distinct clade with a high support value (BP/BS = 1.00/100), and the clade grouped together with *Nalanthamala* species, including *N.vermoesenii*, *N.olivacea* and *N.psidii*. Therefore, two new taxa, *Atractiumyunnanense* sp. nov. and *Nalanthamalaxishuangbannaensis* sp. nov., are proposed according to the phylogenetic analysis.

### ﻿Taxonomy

#### ﻿Sordariomycetes O.E. Erikss. & Winka

**Hypocreales Lindau**

**Nectriaceae Tul. & C. Tul.**

##### ﻿ Atractium

Taxon classificationFungiHypocrealesNectriaceae

Link, Mag. Gesell. naturf. Freunde, Berlin 3(1–2): 10 (1809)

B856153B-F841-56AD-9A48-D4C4195B16FE

≡ Varicosporella Lechat & J. Fourn.

##### ﻿ Atractium yunnanense

Taxon classificationFungiHypocrealesNectriaceae

H. Zheng & Z.F. Yu sp. nov.

F2CDD6EF-0B2A-5796-BE03-245E2A2E8DCB

MB854385

Fig. 2

###### Etymology.

Refers to the Yunnan province where the holotype was collected.

**Figure 2.**
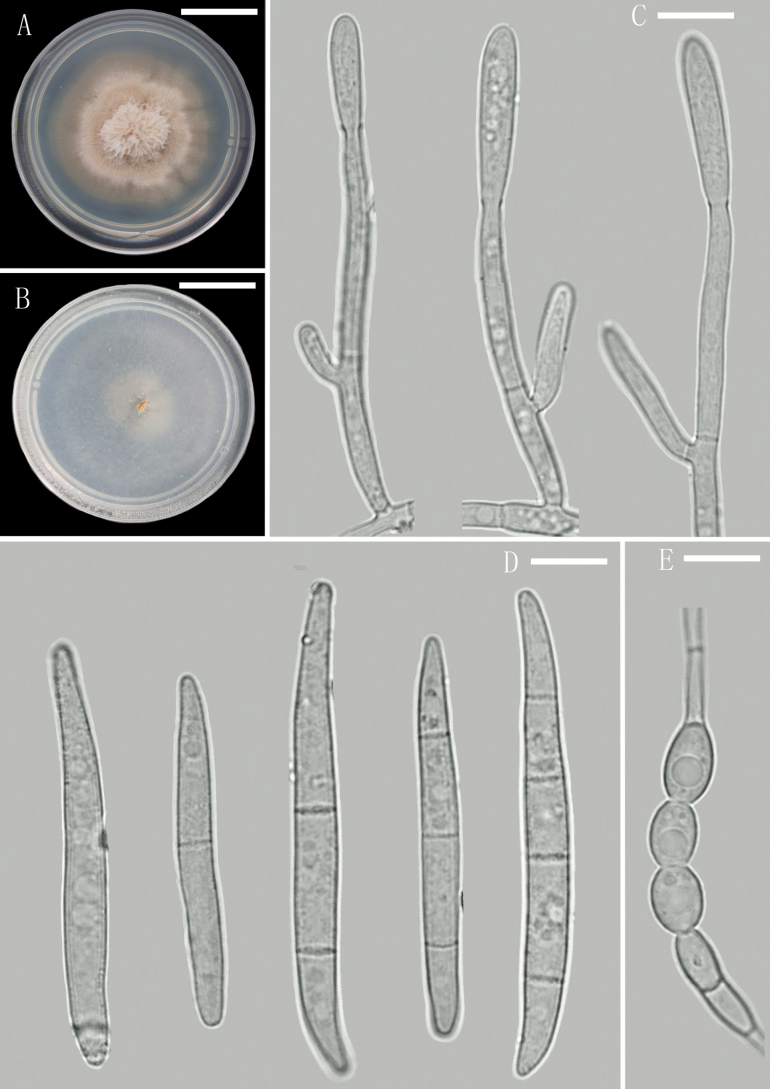
*Atractiumyunnanense* (YMF 1.06524, holotype) **A** colony on PDA after 14 days of inoculation at 28 °C **B** colony on CMA after 3 days of inoculation at 28 °C **C** conidiophores **D** conidia **E** chlamydospores. Scale bars: 1.9 cm (**A, B**); 10 µm (**C–E**).

###### Type.

China • Yunnan province, Huize county; isolated from soil in karst rocky desertification area; Oct 2020; Z.F.Yu, preserved by lyophilization (a metabolically inactive state) in State Key Laboratory for Conservation and Utilization of Bio-Resources in Yunnan (YMF 1.06524, holotype); ex-type living culture: CGMCC 3.20977, other living cultures: GDMCC 3.734; JCM 39337.

###### Description.

Sexual morph not observed. Asexual morph on CMA. ***Hyphae*** 1.5–3 μm wide, pale olivaceous-green, occasionally branched, septate, smooth-walled. ***Conidiophores*** sometimes aggregated into synnemata, nonstromatic, macronematous, mononematous, cylindrical or subulate, straight or flexuous, stipes branched once or twice, monochasial, monoverticillate or irregularly biverticillate, with 1-septate at base. ***Conidiogenous cells*** monophialidic, hyaline, with conspicuous periclinal thickening. ***Conidia*** pale olivaceous-green, solitary, smooth-walled, clavate to oblong-ellipsoidal, slightly curved, with a rounded apical cell, and somewhat conical basal cell, lacking a differentiated foot, 0–3(–4)-septate: 0–1-septate conidia accounting for 8%, 43–65 × 4.5–5.5 μm; 2–3-septate conidia mostly abundant, accounting for 90%, 40.5–67.5 × 4–5.5 μm; 4-septate conidia rare, accounting for 2%, 50.5–57 × 5–5.5 μm. ***Chlamydospores*** 5.5–7.5 × 7–8.5 μm, ellipsoidal, pale olivaceous-green, terminal or intercalary, solitary or in chain.

###### Culture characteristics.

***Colonies*** growing on PDA and CMA after 20 days of incubation at 28 °C. Colony on PDA slow-growing, surface thick, rosy buff to white, reverse white, raised, aerial hyphae abundance, reaching 30–33 mm diam., entire margin. Colonies on CMA flat, surface white, reverse translucent to pale white, aerial hyphae sparsely developed, reaching 40–42 mm diam.

###### Additional materials examined.

China • Yunnan province, Huize county; isolated from soils in karst rocky desertification area; Oct 2020; Z.F.Yu; living cultures H77, H102.

###### Notes.

Phylogenetically, the three strains of *Atractiumyunnanense* (YMF 1.06524, H77 and H102) clustered together in a single clade with a high statistical support (BP/BS = 1.00/100) (Fig. 1). The clade containing *A.yunnanense* and *A.crassum* makes a sister clade to *A.stilbaster*. Morphologically, *A.yunnanense* is similar to *A.crassum* in having clavate to oblong-ellipsoidal and slightly curved conidia. However, the 2–3-septate conidia are most common in *A.yunnanense*, whereas *A.crassum* has most 3–5-septate conidia and no aseptate conidia (Gräfenhan et al. 2011). In addition, the chlamydospores of *A.crassum* are larger and rounder than *A.yunnanense* (7–12 μm diam. vs. 5.5–7.5 × 7–8.5 μm diam.). In a comparison of ITS, LSU and *tub*2 sequences, *A.yunnanense* (type strain YMF 1.06524) has 97% (546/564 bp, 3 gaps), 98% (857/876 bp, 4 gaps) and 95% (403/419 bp, 3 gaps) similarity to *A.crassum* (CBS 180.31), respectively. Moreover, the type species *A.stilbaster* can be morphologically distinguished from *A.yunnanense* in having smaller conidia, 20–40 × 1.5–2.5 μm vs. 40.5–67.5 × 4–5.5 μm (Seifert 1985).

#### ﻿*Nalanthamala* Subram., J. Indian bot. Soc. 35: 478 (1956)

##### ﻿ Nalanthamala xishuangbannaensis

Taxon classificationFungiHypocrealesNectriaceae

H. Zheng & Z.F. Yu sp. nov.

8CF63849-3771-5222-B81F-73AAB447131C

MB854386

Fig. 3

###### Etymology.

Named after the location Xishuangbanna, where the holotype was collected.

**Figure 3.**
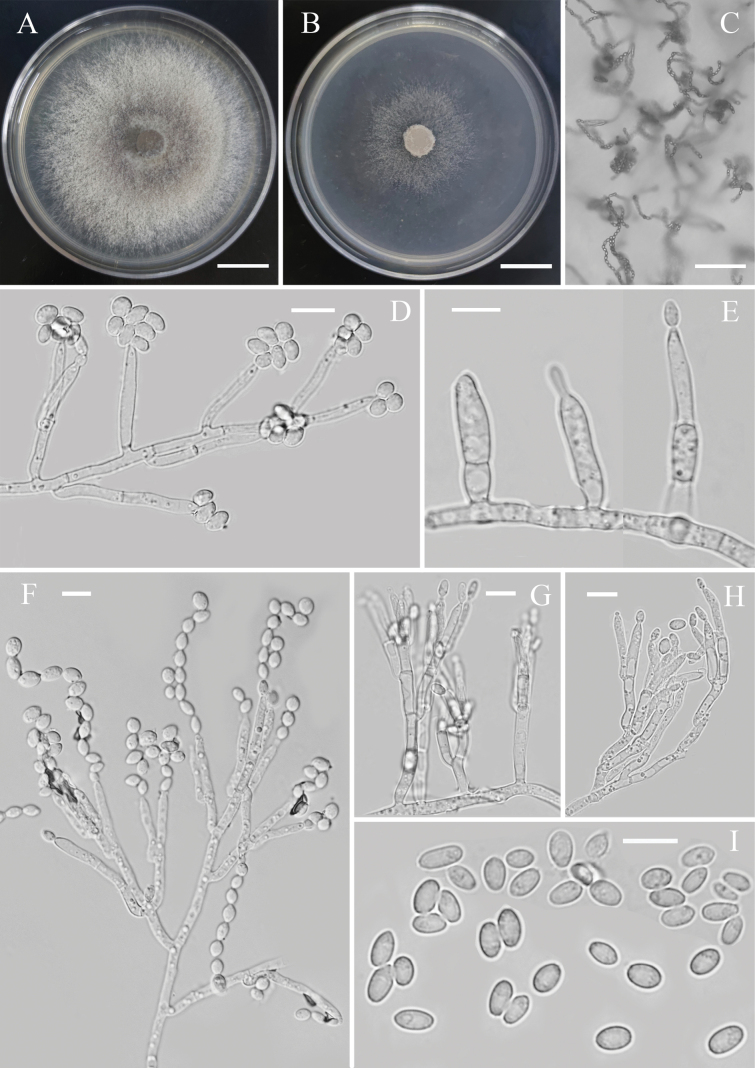
*Nalanthamalaxishuangbannaensis* (YMF 1.05062, holotype) **A** colony on PDA after 3 days of inoculation at 28 °C **B** colony on CMA after 3 days of inoculation at 28 °C **C** conidia arranged in long chains under low power microscopy **D, E** acremonium-like conidiophores **F–H** penicillate conidiophores **I** conidia. Scale bars: 1.0 cm (**A, B**); 50 µm (**C**); 10 µm (**D–I**).

###### Type.

China • Yunnan province, Xishuangbanna Dai Autonomous Prefecture; isolated from air in forest; Apr 2017; Z.F.Yu, preserved by lyophilization (a metabolically inactive state) in State Key Laboratory for Conservation and Utilization of Bio-Resources in Yunnan (YMF 1.05062, holotype); ex-type living culture: CGMCC 3.27596.

###### Description.

Sexual morph not observed. Asexual morph on CMA. ***Hyphae*** 1.5–3.5 μm wide, hyaline, septate, smooth-walled, branched. ***Conidiophores*** hyaline, dimorphic, acremonium-like or penicillium-like. Acremonium-like conidiophores unbranched; phialides cylindrical or slightly tapering toward the tip, straight to slightly bent, 18–37 μm long or longer, 2.3–3.2 μm wide at base, and 1.2–2 μm wide at tip. Penicillium-like conidiophores solitary to aggregated; stipe hyaline, smooth, subcylindrical, septate, 20–80 μm long or longer, up to 3 μm wide at the base; primary and secondary branches subcylindrical, hyaline, smooth, aseptate, 8–16.8 × 2.3–3 µm; phialides cylindrical, flask-shaped, 12.3–22.2 × 2.7–3.2 μm. ***Conidia*** either obovate, with an obtuse tip and a truncated base, typically held in long and dry chains, 4–4.8 × 3–3.8 μm or ellipsoidal, cylindrical, or fusiform, with obtuse ends, or with an obtuse tip and a visible, slightly laterally displaced hilum, 4.4–6 × 2.7–3.5 μm. ***Chlamydospores*** not observed.

###### Culture characteristics.

***Colonies*** growing on PDA and CMA after 3 days of incubation at 28 °C. Colony on PDA rapid-growing, surface dusty to fine powdery, white to iron gray due to occurrence of conidial masses, reverse white, aerial hyphae flocculent, reaching 53–57 mm diam., indistinct margin. Colony on CMA thin, surface white, reverse translucent, aerial hyphae sparsely developed, reaching 35–37 mm diam.

###### Additional materials examined.

China • Yunnan province, Xishuangbanna Dai Autonomous Prefecture; isolated from air in forest; Apr 2017; Z.F.Yu; living cultures B413, B425.

###### Notes.

Morphologically, *Nalanthamalaxishuangbannaensis* fits well with the generic concepts of *Nalanthamala* in having acremonium-like or penicillium-like conidiophores and oval or lenticular conidia arranged in chains (Schroers et al. 2005). In the phylogenetic analysis, the three newly obtained strains (YMF 1.05062, B413 and B425) clustered together in a single clade with a strong statistical support (BP/BS = 1.00/100), and the clade was closely related to *N.diospyri* and *N.psidii* (Fig. 1). *N.xishuangbannaensis* can be distinguished from *N.diospyri* by its wider obovate conidia (3–3.8 μm vs. 2.5 μm in width) and shorter ellipsoidal, cylindrical or fusiform conidia (4.4–6 μm vs. 8–12 μm in length) (Schroers et al. 2005). In a comparison of ITS, LSU and *tub*2 sequences, *N.xishuangbannaensis* (type strain YMF 1.05062) has 95% (469/496 bp, 5 gaps), 97% (691/709 bp, 2 gaps) and 91% (294/323 bp, 3 gaps) similarity to *N.diospyri* (CBS 430.89), respectively. In addition, *N.xishuangbannaensis* differs from *N.psidii* in smaller and wider obovate conidia (4–4.8 × 3–3.8 μm vs. 4.5–5.1 × 2.4–2.7 μm) and shorter and wider ellipsoidal, cylindrical or fusiform conidia (4.4–6 × 2.7–3.5 μm vs. 6–11 × 1.7–2.8 μm) (Schroers et al. 2005). Sequences of *N.xishuangbannaensis* (type strain YMF 1.05062) have 96% similarity in ITS (471/493 bp, 5 gaps) and *tub*2 (329/351 bp, 4 gaps) to *N.psidii* (CBS 687.97).

## ﻿Discussion

Yunnan is uniquely situated at the confluence of three climatic zones: the eastern Asian monsoon zone, the Tibetan Plateau zone, and the tropical monsoon zones of southern Asia and Indochina (Yang et al. 2004). This diverse climatic environment and unique geographical position make the province one of the richest sources of fungi, covering over 40% of the known species in China (Feng and Yang 2018). Nectriaceae species are widespread worldwide, but only one *Atractium* species (*A.fusiformis*) and one *Nalanthamala* species (*N.psidii*) have been recorded from China (Schroers et al. 2005; Gräfenhan et al. 2011; Perera et al. 2023). Therefore, the proposed two new species further showed Yunnan’s high fungi diversity.

Most of Nectriaceae species are soil-borne saprobes or plant pathogens (Lombard et al. 2015; Perera et al. 2023; Chen et al. 2024). Nonetheless, *Atractium* species are commonly associated with water (Gräfenhan et al. 2011). For instance, *A.crassum* was isolated from drinking water in Germany (Gräfenhan et al. 2011), and the three species *A.holubovae*, *A.aquatica* and *A.fusiformis* have been found on submerged decaying wood in the Philippines, France and China respectively (Seifert et al. 1995; Lechat and Fournier 2015; Bao et al. 2023). Only the type species *A.stilbaster* was discovered on bark or stump in Canada and Germany (Gräfenhan et al. 2011). Significantly, our proposed species *A.yunnanense* was discovered from soils in a karst rocky desertification area of Yunnan. This finding extends the habitat of *Atractium* species.

Currently, five species are accepted in *Nalanthamala* (Wijayawardene et al. 2020; Crous et al. 2021). The type species *N.madreeya* was isolated from dead stems in India; unfortunately, the type specimen could not be found in the herbaria and the species has not been recollected. Another four *Nalanthamala* species were reported as associated with wilt and blight diseases from several economically important crops and were more widespread (Schroers et al. 2005). *N.psidii* causes a destructive wilt disease to *Psidiumguajava* trees and is distributed in Malaysia, South Africa, and Taiwan, possibly restricted to subtropical or tropical regions. *N.vermoesenii* could cause necrosis and blight to various Arecaceae species and is distributed in Australia, the Czech Republic, Spain and the Unites States, particularly known from warm temperate, Mediterranean, or (sub)tropical climates. *N.diospyri* could cause a destructive wilt to *Diospyrosvirginiana* and is distributed in the United States. Additionally, *N.olivacea* was isolated from palm in Mexico, Costa Rica, and the Philippines, possibly restricted to tropical regions. Overall, *Nalanthamala* species are mainly distributed in the warm temperate to tropical regions (Schroers et al. 2005). Our proposed new species *N.xishuangbannaensis* was isolated from the air in Xishuangbanna of Yunnan, which is a tropical region, and is also the first *Nalanthamala* species obtained from air.

Despite having cosmopolitan distribution of Nectriaceae species, *Atractium* and *Nalanthamala* species have fewer records in China. Significantly, *A.fusiformis* and *A.yunnanense* were first discovered from Yunnan (Bao et al. 2023). Two *Nalanthamala* species, *N.psidii* and *N.xishuangbannaensis*, were found in China. *N.psidii*, as a widespread species, was found in Taiwan (Rossman et al. 2013), and *N.xishuangbannaensis* was first reported from Yunnan. The discovery of these *Atractium* and *Nalanthamala* species in Yunnan proves once again that it is rich in fungal species. Further studies are needed to understand the pathogenicity of the newly discovered *Atractium* and *Nalanthamala* species.

## Supplementary Material

XML Treatment for Atractium

XML Treatment for Atractium yunnanense

XML Treatment for Nalanthamala xishuangbannaensis

## ﻿Additional information

### Author ORCIDs

Hua Zheng https://orcid.org/0000-0002-0794-6742

Xinwen Dai https://orcid.org/0009-0000-1175-8456

Haiyan Li https://orcid.org/0000-0002-7041-3199

Zefen Yu https://orcid.org/0000-0002-6246-5861

### Data availability

All of the data that support the findings of this study are available in the main text.
